# Does theta synchronicity of sensory information enhance associative memory? Replicating the theta-induced memory effect

**DOI:** 10.1177/23982128241255798

**Published:** 2024-05-24

**Authors:** Fatih Serin, Danying Wang, Matthew H. Davis, Richard Henson

**Affiliations:** 1 MRC Cognition and Brain Sciences Unit, University of Cambridge, UK; 2 Centre for Cognitive Neuroimaging, School of Psychology & Neuroscience, University of Glasgow, UK; 3 Department of Neuroscience, Physiology and Pharmacology, University College London, UK; 4 Department of Psychiatry, University of Cambridge, UK

**Keywords:** Episodic memory, association, binding, multisensory, oscillations, associative memory, theta

## Abstract

The binding of information from different sensory or neural sources is critical for associative memory. Previous research in animals suggested that the timing of theta oscillations in the hippocampus is critical for long-term potentiation, which underlies associative and episodic memory. Studies with human participants showed correlations between theta oscillations in medial temporal lobe and episodic memory. Clouter et al. directly investigated this link by modulating the intensity of the luminance and the sound of the video clips so that they ‘flickered’ at certain frequencies and with varying synchronicity between the visual and auditory streams. Across several experiments, better memory was found for stimuli that flickered synchronously at theta frequency compared with no-flicker, asynchronous theta, or synchronous alpha and delta frequencies. This effect – which they called the theta-induced memory effect – is consistent with the importance of theta synchronicity for long-term potentiation. In addition, electroencephalography data showed entrainment of cortical regions to the visual and auditory flicker, and that synchronicity was achieved in neuronal oscillations (with a fixed delay between visual and auditory streams). The theoretical importance, large effect size, and potential application to enhance real-world memory mean that a replication of theta-induced memory effect would be highly valuable. The present study aimed to replicate the key differences among synchronous theta, asynchronous theta, synchronous delta, and no-flicker conditions, but within a single experiment. The results do not show evidence of improved memory for theta synchronicity in any of the comparisons. We suggest a reinterpretation of theta-induced memory effect to accommodate this non-replication.

## Introduction

Episodic memories often involve multisensory information, but it remains unclear how information from different sensory modalities is bound together. It is commonly argued that episodic memory formation depends on long-term potentiation (LTP; Kandel, 2001; Lynch, 2004). Electrophysiological studies suggest that LTP in the hippocampus is linked with theta frequency oscillations in the same region. Several animal studies showed direct evidence for enhanced and impaired LTP by stimulating the hippocampus at the peak and the trough of theta oscillations, respectively (Hölscher et al., 1997; Huerta and Lisman, 1995; Hyman et al., 2003; Pavlides et al., 1988). In addition, theta oscillations in the human medial temporal lobe (MTL) during encoding have been shown to correlate with episodic memory (Kota et al., 2020; Lega et al., 2012; Staudigl and Hanslmayr, 2013). These findings point to the possibility that theta oscillations influence the formation of episodic memories by enabling LTP to bind together different types of information. Synthesising these lines of work, a recent study by Clouter et al. (2017) devised a non-invasive technique to manipulate brain oscillations in humans during encoding, enabling a direct test of the relationship between binding in episodic memory and brain oscillations.

These authors modulated the intensity of visual and auditory stimuli via a method they called ‘flickering’, and manipulated the phase synchronicity between visual and auditory flickering. The technique periodically modulates the luminance of the video and the amplitude of sound inside the clips at theta frequency (4 Hz). Participants encoded these flickering clips and their memory was later tested by a recognition task in which they had to distinguish studied from unstudied clips. The clips that had synchronous audio and video flicker during encoding were more accurately recognised compared to asynchronous clips. In two follow-up experiments, theta synchronicity was compared with non-flickering clips and to synchronicity at alpha and delta frequencies. Participants’ memory was better for clips that had synchronous theta modulation than if audio and video streams were unmodulated or were synchronously modulated at alpha (10.5 Hz) and delta (1.7 Hz) frequencies. Based on these results, Clouter et al. (2017) concluded that theta-phase synchronicity facilitates the binding of information from different modalities in episodic memory, which they call the theta-induced memory effect (TIME). A subsequent study by the same group (Wang et al., 2018) replicated the better memory performance in a synchronous theta condition compared to an asynchronous condition, and furthermore used electroencephalography (EEG) to show that performance related to the phase difference between entrained neural activity in visual and auditory cortices. However, this study did not include a no-flicker condition or conditions containing other flicker frequencies.

TIME has considerable theoretical implications. Indeed, computational modelling by Wang et al. (2023) shows how spike-time-dependent plasticity (STDP) predicts this benefit of synchronous input to MTL at theta frequency. TIME also has potential practical applications. For example, theta modulation of stimuli might help memory performance (e.g. for people with memory problems). Despite the theoretical and practical importance of TIME, there has not yet been a replication of the combined behavioural results of Clouter et al. (2017) and Wang et al. (2018) outside the original research group. Of particular importance for interpretation of memory enhancement effects is a replication in which all relevant control conditions (asynchronous theta, no-flicker, and synchronous non-theta) are combined in a single experiment. This is the aim of the present experiment.

We used the same stimuli and methods as in Clouter et al. (2017), with four conditions: (1) synchronous theta, (2) asynchronous theta, (3) synchronous delta, and (4) no-flicker. Note that we proposed to use delta rather than alpha as the control frequency given some suggestion in Clouter et al.’s (2017) study that alpha modulation improves memory regardless of synchronicity. Finding better memory in the synchronous theta condition than all three other conditions would confirm the specificity of the effect to synchronous theta, supporting the interpretations offered by Clouter et al. (2017).

However, we proposed two small procedural modifications. The first concerns the duration of the stimuli at encoding. Clouter et al. (2017) used shortened clips for the no-flicker condition (1.5 s instead of 3 s) in an attempt to match the total amount of information (i.e. integrated amplitude of the visual/auditory stimuli across time, given that this amplitude varies sinusoidally in the flicker conditions). However, it may be that memory is positively related to the duration of the clips, which could explain why Clouter et al. (2017) found worse memory for the no-flicker stimuli. Therefore, we proposed to match the total duration of the clips across all conditions. To maintain the total amplitude across conditions, no-flicker stimuli had video and sounds with halved (50%) amplitude. One possible outcome is that, by controlling duration, we will now observe better memory in the no-flicker condition than synchronous theta condition, for example if flickering of any type is generally distracting and impairs memory. This would require a radical re-interpretation of TIME, since it would suggest that synchronous theta is less distracting than other types of flicker, but does not benefit memory (or STDP) per se.

A second procedural change is that we presented the synchronous and asynchronous theta conditions in different blocks, whereas in the previous experiments, synchronous and asynchronous trials were intermixed within the same block. It is important not to intermix flicker with no-flicker trials, in case entrainment effects from flicker trials carry over into subsequent no-flicker trials. The same concern of carry-over effects could arise if intermixing flicker trials at different frequencies. Given that our no-flicker and synchronous delta conditions cannot be intermixed, it is best to block all conditions. It is possible that the memory difference between synchronous and asynchronous theta trials will be lost when blocked, for example if the difference arises from the contrast between closely occurring trials (e.g. if participants find synchronous trials more pleasant, they may devote more attention to these trials than surrounding asynchronous ones). Given that Clouter et al. and Wang et al. also found that their participants could not explicitly detect the perceptual difference between synchronous and asynchronous theta trials, this account seems unlikely, but it remains a possibility. However, we acknowledge that, should one or both of these procedural changes mean that we do not replicate the findings of Clouter et al. (2017), we would have to run further experiments to establish which procedural deviation is important for replicating TIME.

In summary, Clouter et al. (2017) demonstrated that theta synchronicity improves multimodal memory in humans; an effect that they called TIME. Due to the theoretical and practical importance of this effect, we think it would be prudent for an independent group to conduct a replication of TIME. We proposed to do so by testing the critical test and control conditions within a single group of participants, and with the two small procedural variations described above to rule out potential alternative explanations.

## Methods

Approved Stage 1 protocol for this study can be found in Open Science Framework PsyArXiv: https://osf.io/preprints/psyarxiv/unprw

### Sample

We conducted a power analysis using G*Power (Faul et al., 2009). For the effect size of interest, we chose the smallest effect size (Cohen’s *d* = 0.66) among the comparisons from Clouter et al.’s (2017) study and Wang et al.’s (2018) study. To acquire 90% statistical power for three, one-tailed, paired *t*-tests with alpha = .0167 (corrected for three comparisons), 30 participants are required. However, for counterbalancing purposes (see below), we recruited 32 participants, which provides 92.8% statistical power. The participants were young adults (25 females, mean age = 25.59 years, standard deviation (*SD*) = 3.23, range = (21–34 years)) from Cambridge, UK residents from the volunteer panel of the MRC Cognition and Brain Sciences Unit. Participants were excluded from recruitment according to history of photosensitivity, given that the experiment involves rapidly flickering visual stimuli. Participants’ overall memory performance was tested against chance level (25%) according to non-parametric permutation. No participants were excluded with this criterion. They provided informed consent and received monetary compensation. Participants had self-reported normal hearing and correctable vision.

### Stimuli

The stimuli and the scripts for running the experiments are adapted from Clouter et al.’s (2017) study as provided by the authors. Each movie clip is 3 s long and is created by randomly matching videos and sounds from the stimulus pool. The stimulus pool was assembled such that the videos and sounds would not be semantically connected. The flickering was generated by MATLAB scripts via sinusoidal modulation of visual and auditory intensities. The modulation starts at 50% amplitude, and then, alternates between 100% and 0% amplitudes in sinusoidal manner. The clips for the no-flicker condition will be presented with 50% amplitude, meaning that amplitudes of the video and sound streams are be halved. This procedure matches the total amplitude between the flickering and non-flickering clips while also having matching durations.

### Procedure

Data collection took place in a sound-attenuating and magnetically shielded room (MSR), while participants are seated beneath a MEGIN Triux MEG scanner. MEG data will be acquired simultaneously, with the aim of confirming differences across conditions in the synchronicity of MEG responses observed in visual and auditory cortices (as Clouter et al. and Wang et al. did for EEG). However, these MEG data are not part of the behavioural analysis that we are registering here. Instructions and visual stimuli were projected onto a screen through an aperture in the front wall of the MSR. Participants were given MEG-compatible glasses to correct their vision. Auditory stimuli were presented binaurally via MEG-compatible headphone drivers (Etymotic Research, https://www.etymotic.com) sending audio signals down plastic tubes to ear plugs inserted in participants’ ears. The visual and auditory delays between the stimulus presentation computer and participants’ eyes/ears were confirmed by a photo-diode and microphone using a standard procedure employed in the CBU MEG laboratory.

**Figure 1.**
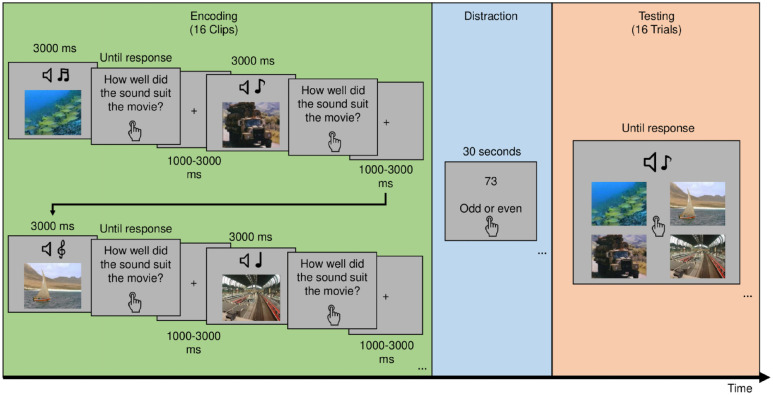
Depiction of the typical experimental flow in a block. Participants are first presented with a series of clips that are either modulated or not depending on which condition is assigned to that block. After each clip, they rate how well the sound suited the video. Then, they count backwards from a random number. Finally, they are tested on these clips by being presented with the sound and asked to pick the video associated with it.

The experiment consists of 12 blocks, with three tasks per block, and with each block containing trials from one condition only (Figure 1). In Task 1, participants were presented with the 3000-ms movie clips (subtending a visual angle of 5.7°) and after each clip, they were asked to rate the compatibility of the sound and video to encourage paying attention to both modalities. Each rating was followed by a fixation cross on a blank screen for an inter-stimulus interval that is jittered between 1000 and 3000 ms. The clips either flickered synchronously at theta rate (4 Hz), asynchronous at theta, synchronous at delta (1.7 Hz), or non-flickering. In Task 2, participants were instructed to make odd–even judgement for random numbers appearing on the screen to distract them from rehearsing. In Task 3, participants were presented with a recognition test. In each trial, one of the sounds presented in Task 1 was played and the participants were asked to pick the associated video from four stills, each from a different studied video in the same block. There were 16 test trials to test every clip presented in Task 1. Each block included four sound categories out of eight, and each clip was tested within its own category to better assess associative memory.

The video and sound stimuli were randomly paired, and then the pairs divided into four sets, whose assignment to conditions was rotated across participants. Given the 24 possible orderings of four conditions (blocks), this order was counterbalanced by presenting three unique orderings for each of every eight participants (e.g. the 12 blocks of Participant 1 corresponded to conditions SADN-ADNS-DNSA, the 12 blocks of Participant 2 was NSAD-ASDN-SAND, etc.; where S = synchronous theta, A = asynchronous theta, D = delta, and N = no flicker). Thus, full counterbalancing entailed 4 × 8 = 32 participants.

Following the main experiment, participants performed a discrimination task that assessed their ability to discern synchronous stimuli from asynchronous stimuli. This involved blocked presentation of random synchronous or asynchronous clips, each followed by a judgement of synchronicity. Blocking the conditions makes this task mimic the main task which would provide a better measurement of whether participants were able to tell synchronous from asynchronous stimuli during main task. However, it should be noted that the decision was taken after the approval of Stage 1 protocol.

The synchronicity above refers to synchronicity of information processed between the relevant cortices, rather than synchronicity of the stimuli themselves. Following Clouter et al. (2017) and Wang et al. (2018), to account for the slower transfer of visual information to visual cortex than of auditory information to auditory cortex, the onset of the auditory stimuli was delayed by 40 ms (though note this does not allow for potential differences in transmission time for information from visual versus auditory cortex to hippocampus). An analysis of the phase differences in recordings of the visual and auditory stimulus channels (sampled at 1 kHz and stored with the MEG data) confirmed that delays were close to those intended (see the Supplemental Material for details).

### Statistical analysis

We performed the main analysis on single-trial data (correct/incorrect), using a logistic mixed-effects model to gain greater sensitivity and accommodate variability across participants and stimulus pairs. We added random slopes and intercepts for both participants and stimuli, which achieved convergence (Barr et al., 2013). Within this model, we calculated *p*-values for the three planned comparisons: (1) synchronous theta against asynchronous theta, (2) synchronous theta against synchronous delta, and (3) synchronous theta against no-flicker (predicting higher accuracy for synchronous theta conditions in all cases). Due to three comparisons, we used an adjusted, one-tailed significance threshold of α_corrected_ = .0167. The analyses will be run in R (R Core Team, 2020), using the *lme4* package (Bates et al., 2015).

However, to be able to report statistical results comparable to Clouter et al.’s (2017) study, and to match the power analysis on which our sample size was determined, we also performed one-tailed, *t*-tests on trial-averaged data for the same three planned comparisons listed above (i.e. synchronous theta against each of the control conditions). We report both asymptotic *p*-values and percentile bootstrapped probabilities.

## Results

All statistical analyses were run using *R* version 4.2.2 in *RStudio* version 2022.12.0+353, using *lme4* package version 1.1-33 for the mixed-effects model, and *emmeans* version 1.8.6 for contrasts with estimated marginal means, Package *MKinfer* version 1.1 for *t*-tests with bootstrapping. The code and data are available on GitHub (https://github.com/fserin/ReplicatingTIME.git)

### Confirmatory analyses

**Figure 2.**
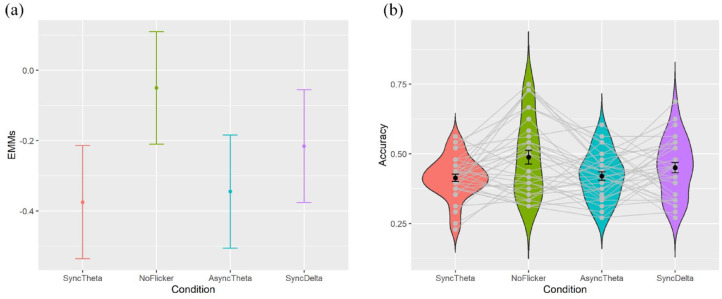
(a) Plots the estimated marginal means (log odds) from the registered mixed-effects model for each condition. (b) Plots the raw trial-averaged means for each condition. Error bars show 95% confidence intervals of the group-level variance.

Participants had an overall accuracy of *M* = 44.3% (*SD* = 7.15%; chance = 25%) and for only synchronous and asynchronous theta conditions *M* = 41.4%, which compares reasonably to the mean performance of 46.4% and 42.8% reported by Clouter et al. (2017) and Wang et al. (2018), respectively. According to the registered analysis, we first fit a binary logistic mixed-effects model to every trial with the following form:

Accuracy~Condition+(1|ClipID)+(1|ParticipantID),

where Accuracy was the binary outcome for each trial, Condition was a categorical fixed effect with four levels, and ClipID and ParticipantID were modelled as random intercepts. There was a significant difference between conditions, *X*^2^(3) = 22.9, *p* < .0001. In our three planned comparisons, we predicted better performance for the synchronous theta condition than each of the other three conditions. However, planned contrasts on the estimated marginal means (Figure 2(a)) showed that the synchronous theta condition actually produced worse memory performance than the no-flicker condition, *Z* = −4.29, *p* < .0001, log odds effect size = 0.33. The synchronous theta condition did not differ significantly from the asynchronous theta condition, *Z* = −0.393, *p* = .695, log odds effect size = −0.03. Finally, the synchronous theta condition also had worse rather than better performance than the synchronous delta condition, *Z* = −2.10, *p* = .0355, log odds effect size = −0.159, though the latter *p*-value did not survive our registered corrected threshold of α_corrected_ = .05/3 = .0167.

To match Clouter et al. (2017), we also report the analysis of trial-averaged accuracy scores (Figure 2(b)) using paired *t*-tests and bootstrapped *p*-values. Similar to the mixed-effects results, the synchronous theta condition (*M* = 41.4%) had worse performance than the no-flicker condition (*M* = 48.8%), *t*(31) = −3.09, *p* < .004, Cohen’s *d* = 0.55, but did not differ significantly from the asynchronous theta condition (*M* = 42.02%), *t*(31) = −0.48, *p* = .614, Cohen’s *d* = 0.08, nor synchronous delta condition (*M* = 45.05%), *t*(31) = −1.72, *p* = .082, Cohen’s *d* = 0.30.

We also report the *d*-prime score for the performance in the synchrony discrimination task. Correctly identified synchronous trials were treated as hits and asynchronous trials mistaken as synchronous were treated as false alarms. This eliminates any confounding effects of response bias. Unfortunately, due to an error in condition files, nine participants did not have usable data for the discrimination task and therefore, the data could not be reported for these. When *d*-primes were compared against zero using a two-tailed, one-sample *t*-test, the mean *d*-prime was not significantly above chance, *t*(22) = 2.23, *p* = .0518, producing no evidence people could perceive the difference between synchronous and asynchronous stimuli (see also the Exploratory analyses section). This replicates Clouter et al. (2017), even when these two conditions are blocked. However, considering the small *p*-value and the few missing participants from the dataset, it is possible that some participants were able to detect synchronicity.

### Exploratory analyses

We report additional post hoc, exploratory analyses that might be helpful to better understand the boundary conditions for any TIME. Through personal communication, it was reported that proactive interference on memory performance is possible due to the recurring sound categories across blocks. To test this, we analysed only the first of the 12 blocks for each participant using the same mixed-effects model. This analysis failed to reproduce the significantly worse performance for synchronous theta versus no-flicker conditions, *Z* = −0.76, *p* = .447, though the direction remained the same (and the lack of significance could reflect the vastly reduced power). The outcomes for the other two comparisons did not differ in either significance or direction of the numerical difference. To complement this, we also added a linear and quadratic expansion of block number to the mixed-effects model, but while there were main effects of block (e.g. due to practice, fatigue, interference), *X*^2^(3) = 14.85, *p* = .0006, there was no evidence that these effects interacted with the condition factor *X*^2^(6) = 22.9, *p* = .948.

It was also suggested that using the overall accuracy to exclude participants might have missed participants who performed specifically worse in theta conditions. To evaluate this, we used only the accuracy in theta conditions (both synchronous and asynchronous) to exclude participants. Three participants were excluded as a result. However, the pattern of significant outcomes did not differ from the registered ones above (synchronous theta versus no-flicker, *Z* = −3.61, *p* = .0003, synchronous theta versus asynchronous theta and synchronous delta,|*Z|*s < −1.63, *p*s > .105).

It is probable that the ratings (the subjective match between video and sound) of the clips during the initial encoding task differed across conditions, and that these ratings influenced memory performance. Applying the three planned comparisons to a mixed-effect model that predicted the rating score from the condition factor (with random intercepts for ClipID and ParticipantID) revealed that the synchronous theta condition was rated significantly less matching than the no-flicker condition, *Z* = −4.60, *p* < .0001, but did not differ significantly for either the asynchronous theta or the synchronous delta conditions, |*Z|*s < −0.391, *p*s > .696. We therefore included a linear and quadratic expansion of the rating scores for every trial in an augmented version of the main mixed-effect model that predicted accuracy for each trial. While there was a significant main effect of rating on memory, *X*^2^(2) = 85.8, *p* < .0001, with more matching trials being more likely to be remembered, this did not interact significantly with the condition factor, *X*^2^(6) = 10.1, *p* = .12. More importantly, the three planned comparisons on memory accuracy as a function of pairs of conditions showed the same pattern of significant results (synchronous theta versus no-flicker, *Z* = −3.49, *p* = .0005, synchronous theta versus asynchronous theta and synchronous delta,|*Z|*s < −1.92, *p*s > .055).

Finally, even though the mean discrimination of synchronous and asynchronous theta trials did not differ significantly from zero (see the Results section), we correlated synchrony judgement ratings with memory accuracy, to see if the perception of synchronicity influenced memory (e.g. if a subset of participants could detect above chance). Since *d*-prime only exists at the participant level, we performed a simple Pearson correlation across participants, for which a *t*-test showed no evidence that the slope was greater than zero, *t*(21) = 1.41, *p* = .172 (see the Supplemental Material for details). However, we acknowledge that the power of this statistical test is low, particularly given that we did not collect perceptual discriminability for the full sample of participants.

## Discussion

Our planned comparisons on memory for audio–visual associations showed no benefit of synchronous theta flickering at encoding, compared to any of the asynchronous theta, synchronous delta, or no-flicker conditions, whether in terms of the estimated marginal means of the single-trial mixed-effects model, or as *t*-tests on trial-averaged data. In fact, the planned comparison against our no-flicker condition showed worse performance, rather than the better performance reported by Clouter et al. (2017). The most likely reason for the latter is that our no-flicker condition matched the duration of trials at encoding (as well as the mean intensity), whereas the no-flicker trials in Clouter et al. (2017) were half as long as the flicker trials, and it seems likely that shorter durations of stimuli at encoding would lead to poorer memory. Together with the lack of evidence for any advantage of synchronous theta against asynchronous theta or synchronous delta, we conclude that we have failed to replicate the TIME reported by Clouter et al. (2017) and Wang et al. (2018).

The second major change we made to the experimental procedure of Clouter et al. (2017) was to block all conditions, rather than intermix synchronous and asynchronous theta trials. The reasons for this change were given in the Introduction section, and it remains possible that the contrast between changing synchronicity across trials is important for the TIME to be seen. We cannot rule out this possibility, though we note that even with our blocking of these two conditions, participants’ ability to consciously detect synchronicity between the auditory and visual streams was, like Clouter et al. (2017), not statistically different from chance. Future studies could explicitly compare intermixed and blocked synchronous and asynchronous conditions.

It is worth noting that we did not quite achieve the intended 40-ms delay between auditory and visual stimuli for the synchronous theta condition. This target of 40 ms was based on the original authors’ estimate of the earliest ERP in auditory cortex being ~10 ms (the ‘middle latency response’) whereas the earliest ERP for visual cortex (the ‘C1’) being ~50 ms. Recordings of our stimuli suggested that the auditory theta envelope only preceded the visual one by ~25 ms (after taking into account sound delay from the air-tubes, see the Supplemental Material), meaning that neural activity in visual cortex may have been ~15 ms later than intended (or ~20° of theta phase angle). We do not think this difference is likely to be important however, given the uncertainty (and likely individual differences) in the above estimates of neural delays. Nonetheless, future studies could aim for a neural phase difference of exactly 0°.

We performed some additional exploratory analyses to investigate other factors that might affect the ability to detect a TIME. However, the pattern of significant results, or at least conclusions one would draw, was unchanged when we: (1) analysed the first block only, to reduce interference effects across trials from using similar auditory stimuli from the same categories, (2) only included participants whose memory was above chance in the theta conditions alone (as opposed to the registered criterion of average over all conditions), (3) statistically adjusted for match ratings at encoding, which did differ between flicker and no-flicker conditions, and (4) correlated the difference in memory between synchronous minus asynchronous conditions with individual variability in participants’ ability to discriminate these conditions perceptually, for which this correlation failed to reach significance.

## Conclusion

Episodic memories often bind representations from multiple sensory modalities. Clouter et al. (2017) focused on this aspect of episodic memories to understand how information from different sensory sources might be bound together in the brain. Following on from previous animal research that directly linked theta frequency hippocampal activity to LTP, and human research that demonstrated links between hippocampal theta and subsequent memory performance, Clouter et al. (2017) provided direct evidence for the role of theta frequency activity in the human brain, by modulating the amplitude of visual and audio pairings in the theta band. This ‘TIME’ promises methodological, theoretical, and practical benefits. In the present work, we attempted to replicate this TIME, as well as raising alternative explanations that could potentially result in its reinterpretation. Unfortunately, we found no evidence for this effect, along with evidence for alternative explanations (e.g. for why their no-flicker baseline may have underestimated memory by not controlling for stimulus duration). Nonetheless, we are aware of the potential impact of some of the procedural changes that we registered, and so plan future experiments with closer replications, for example to examine intermixed rather than blocked synchronous and asynchronous trials.

## Supplemental Material

sj-docx-1-bna-10.1177_23982128241255798 – Supplemental material for Does theta synchronicity of sensory information enhance associative memory? Replicating the theta-induced memory effect Supplemental material, sj-docx-1-bna-10.1177_23982128241255798 for Does theta synchronicity of sensory information enhance associative memory? Replicating the theta-induced memory effect by Fatih Serin, Danying Wang, Matthew H. Davis and Richard Henson in Brain and Neuroscience Advances

## References

[bibr1-23982128241255798] Barr DJ Levy R Scheepers C , et al. (2013) Random effects structure for confirmatory hypothesis testing: Keep it maximal. Journal of Memory and Language 68(3): 255–278. 10.1016/j.jml.2012.11.001 PMC3881361 24403724

[bibr2-23982128241255798] Bates D Mächler M Bolker B , et al. (2015) Fitting linear mixed-effects models using lme4. Journal of Statistical Software 67(1): 1–48.

[bibr3-23982128241255798] Clouter A Shapiro KL Hanslmayr S (2017) Theta phase synchronization is the glue that binds human associative memory. Current Biology 27(20): 3143–3148.e6. 10.1016/j.cub.2017.09.001 28988860

[bibr4-23982128241255798] Faul F Erdfelder E Buchner A , et al. (2009) Statistical power analyses using G*Power 3.1: Tests for correlation and regression analyses. Behavior Research Methods 41(4): 1149–1160. 19897823 10.3758/BRM.41.4.1149

[bibr5-23982128241255798] Hölscher C Anwyl R Rowan MJ (1997) Stimulation on the positive phase of hippocampal theta rhythm induces long-term potentiation that can be depotentiated by stimulation on the negative phase in area CA1 in vivo. The Journal of Neuroscience 17(16): 6470–6477. 9236254 10.1523/JNEUROSCI.17-16-06470.1997 PMC6568346

[bibr6-23982128241255798] Huerta PT Lisman JE (1995) Bidirectional synaptic plasticity induced by a single burst during cholinergic theta oscillation in CA1 in vitro. Neuron 15(5): 1053–1063. 7576649 10.1016/0896-6273(95)90094-2

[bibr7-23982128241255798] Hyman JM Wyble BP Goyal V , et al. (2003) Stimulation in hippocampal region CA1 in behaving rats yields long-term potentiation when delivered to the peak of theta and long-term depression when delivered to the trough. The Journal of Neuroscience 23(37): 11725–11731. 14684874 10.1523/JNEUROSCI.23-37-11725.2003 PMC6740943

[bibr8-23982128241255798] Kandel ER (2001) The molecular biology of memory storage: A dialogue between genes and synapses. Science 294(5544): 1030–1038. 11691980 10.1126/science.1067020

[bibr9-23982128241255798] Kota S Rugg MD Lega BC (2020) Hippocampal theta oscillations support successful associative memory formation. The Journal of Neuroscience: The Official Journal of the Society for Neuroscience 40(49): 9507–9518. DOI: 10.1523/JNEUROSCI.0767-20.2020; https://www.jneurosci.org/content/40/49/9507.abstract 10.1523/JNEUROSCI.0767-20.2020 PMC7724134 33158958

[bibr10-23982128241255798] Lega BC Jacobs J Kahana M (2012) Human hippocampal theta oscillations and the formation of episodic memories. Hippocampus 22(4): 748–761. 21538660 10.1002/hipo.20937

[bibr11-23982128241255798] Lynch MA (2004) Long-term potentiation and memory. Physiological Reviews 84(1): 87–136. 14715912 10.1152/physrev.00014.2003

[bibr12-23982128241255798] Pavlides C Greenstein YJ Grudman M , et al. (1988) Long-term potentiation in the dentate gyrus is induced preferentially on the positive phase of θ-rhythm. Brain Research 439(1–2): 383–387. 3359196 10.1016/0006-8993(88)91499-0

[bibr13-23982128241255798] R Core Team (2020) R: A language and environment for statistical computing. R Foundation for Statistical Computing. Available at: https://www.R-project.org/

[bibr14-23982128241255798] Staudigl T Hanslmayr S (2013) Theta oscillations at encoding mediate the context-dependent nature of human episodic memory. Current Biology 23(12): 1101–1106. 23746635 10.1016/j.cub.2013.04.074

[bibr15-23982128241255798] Wang D Clouter A Chen Q , et al. (2018) Single-trial phase entrainment of theta oscillations in sensory regions predicts human associative memory performance. The Journal of Neuroscience 38(28): 6299–6309. 29899027 10.1523/JNEUROSCI.0349-18.2018 PMC6596103

[bibr16-23982128241255798] Wang D Parish G Shapiro KL , et al. (2023) Interaction between theta phase and spike timing-dependent plasticity simulates theta-induced memory effects. eNeuro 10(3): 1–43. 10.1523/ENEURO.0333-22.2023 PMC10012328 36810147

