# Supplementary material for: Does theta synchronicity of sensory information enhance associative memory? Replicating the theta-induced memory effect
Source: Brain Neurosci Adv. 2024 May 24;8:23982128241255798. doi: 10.1177/23982128241255798 (PMC11127570; doi:10.1177/23982128241255798)
Supplement: sj-docx-1-bna-10.1177_23982128241255798 – Supplemental material for Does theta synchronicity of sensory information enhance associative memory? Replicating the theta-induced memory effect [file sj-docx-1-bna-10.1177_23982128241255798.docx]

# Supplementary Material for

“Does Theta Synchronicity of Sensory Information Enhance Associative Memory? Replicating the Theta-Induced Memory Effect”

By Fatih Serin, Danying Wang, Matthew H. Davis, and Richard Henson

## Figure of the Phase Difference Analysis of the MEG Stimulus Channels


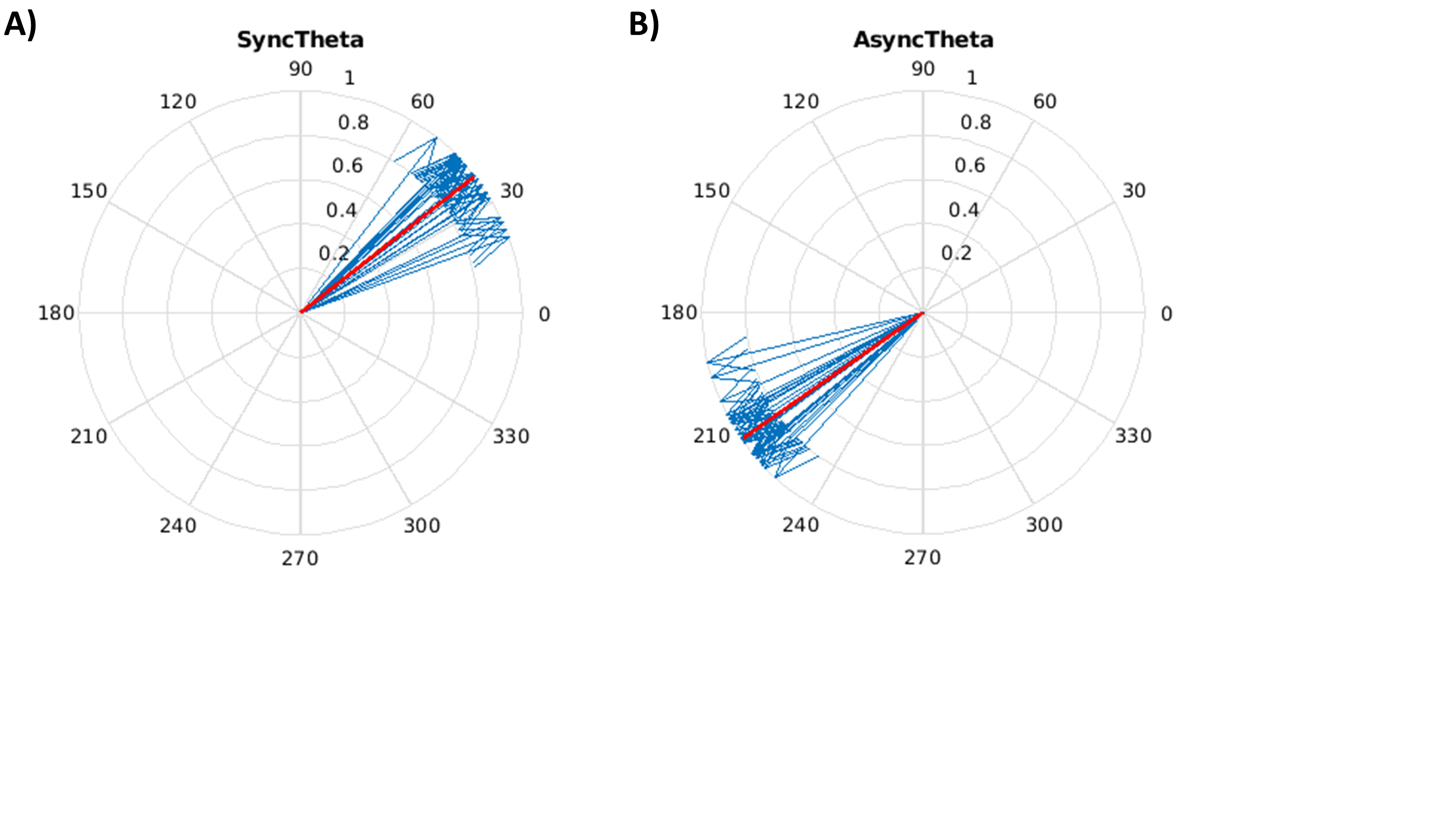


The compass plots show the phase difference between the visual (continuous photodiode) and auditory (audio output recorded into MEG) channels recorded at 1 kHz along with the MEG. The histograms are plotted separately for the conditions: synchronous theta (**A**), and asynchronous theta (**B**). Because the audio recording did not take into account the time for the sound to travel through the tubes to reach the participant in the MEG, 10ms was added to the beginning of the sound signal after calculating the delay in the tubes (3.5 meters). Phase differences are calculated by first taking the envelope of the audio output, bass-pass filtering the data to 4 Hz, Hilbert transforming the data get the angle information, and subtracting the phase of audio signal from the visual signal. The synchronous theta condition showed a mean auditory signal delay of ~25.5 ms / 36.7° at 4 Hz. This is slightly lower than the intended 40 ms / 57° delay, however, the collaborator from the original research group interpreted it as within reasonable range to be considered as synchronous. This 40 ms / 57° shift was intended to allow for slower cortical processing of visual than auditory information (considering the earliest evoked responses in each sensory cortex; see Clouter et al., 2017), and the asynchronous condition has a phase difference of 180-57=123° as a consequence.

## Figure of D-prime Scores against TIME Effect

##
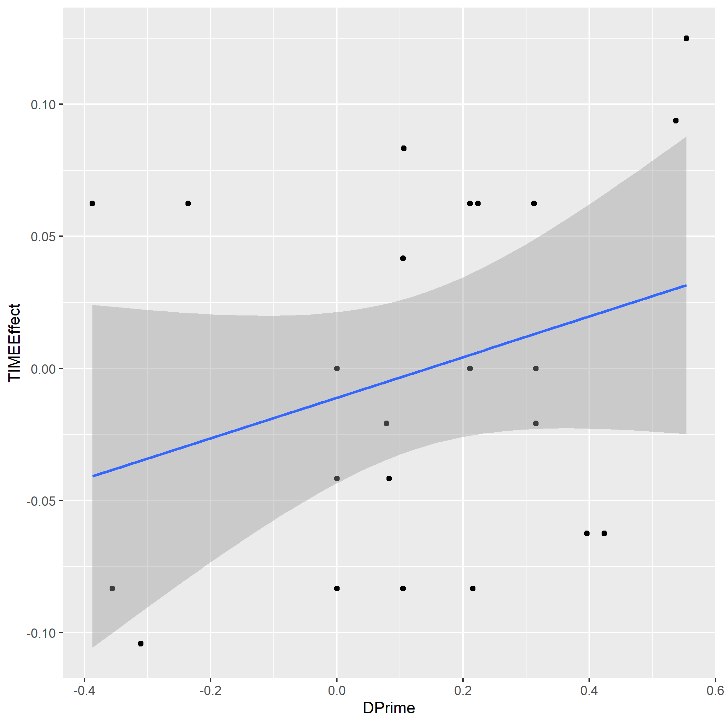


The figure plots the d-prime scores for the synchronicity discrimination task against the TIME scores, which are calculated by subtracting the asynchronous theta accuracy from synchronous theta accuracy for each participant. Therefore, higher scores on y-axis reflect better memory performance in synchronous theta condition in comparison to performance in asynchronous theta condition. There is a trend for a positive relationship, but the correlation does not reach significance (see main paper).

**References**

Clouter, A., Shapiro, K. L., & Hanslmayr, S. (2017). Theta Phase Synchronization Is the Glue that Binds Human Associative Memory. *Current Biology*, *27*(20), 3143-3148.e6. https://doi.org/10.1016/j.cub.2017.09.001
